# PM_2.5_ Extracts Induce INFγ-Independent Activation of CIITA, MHCII, and Increases Inflammation in Human Bronchial Epithelium

**DOI:** 10.3390/toxics12040292

**Published:** 2024-04-16

**Authors:** Héctor Jirau-Colón, Braulio D. Jiménez-Vélez

**Affiliations:** 1 Department of Biochemistry, University of Puerto Rico Medical Sciences Campus, San Juan 00935, Puerto Rico; hector.jirau@upr.edu; 2 Center for Environmental and Toxicological Research, Biochemistry Department, San Juan 00935, Puerto Rico

**Keywords:** particulate matter, respiratory toxicology, inflammation, CIITA, Puerto Rico

## Abstract

The capacity of particulate matter (PM) to enhance and stimulate the expression of pro-inflammatory mediators has been previously demonstrated in non-antigen-presenting cells (human bronchial epithelia). Nonetheless, many proposed mechanisms for this are extrapolated from known canonical molecular pathways. This work evaluates a possible mechanism for inflammatory exacerbation after exposure to PM_2.5_ (from Puerto Rico) and CuSO_4_, using human bronchial epithelial cells (BEAS-2B) as a model. The induction of CIITA, MHCII genes, and various pro-inflammatory mediators was investigated. Among these, the phosphorylation of STAT1 Y701 was significantly induced after 4 h of PM_2.5_ exposure, concurrent with a slight increase in CIITA and HLA-DRα mRNA levels. INFγ mRNA levels remained low amidst exposure time, while IL-6 levels significantly increased at earlier times. IL-8 remained low, as expected from attenuation by IL-6 in the known INFγ-independent inflammation pathway. The effects of CuSO_4_ showed an increase in HLA-DRα expression after 8 h, an increase in STAT1 at 1 h, and RF1 at 8 h We hypothesize and show evidence that an inflammatory response due to PM_2.5_ extract exposure in human bronchial epithelia can be induced early via an alternate non-canonical pathway in the absence of INFγ.

## 1. Introduction

Particulate matter (PM) has been recognized as a global health problem, partially responsible for the exacerbation of many adverse health effects, particularly those associated with airway inflammation. Estimates of seven million people die due to air pollution every year, according to the World Health Organization (WHO) [1]. The WHO has recommended a standard permissible level of air contaminants, but nearly 80% of urban cities are well above it [2]. Airborne PM is a complex mixture of small particles (PM_10_, PM_2.5_, PM_<1_) enriched with inorganic and organic constituents from variable sources. The high variability in PM composition depends not only on location but also on time (seasons). Public health studies have concentrated on airborne PM, given the concern of its effect due to rapid population growth (industrialization and urbanization) and increasing anthropogenic emissions modifying its content [3]. Epidemiological evidence links PM with type 2 diabetes mellitus (T2DM) increased risk of diabetes, fasting plasma glucose (FPG), and glycosylated hemoglobin (HbA1c) [4]. Most recently, evidence of the temporal association between long-term PM_2.5_ exposure and higher FPG and incident T2DM in two cities’ environments in India was reported [5].

It has been reported that airborne PM_2.5_ induces adverse health problems such as decreased lung function, exacerbation of asthma, chronic obstructive pulmonary disease (COPD), and cardiovascular outcomes triggered by the particle itself or its constituents [6,7,8,9]. Likewise, PM_2.5_ exposure could also be associated with other health-related problems, including pre-term birth, lung development in children, and reduced lung growth rate [8,10] as well as diabetes [11,12].

Understanding the mechanism by which PM constituents exacerbate health problems has been a topic of great concern, debate, and discussion. Inhalation toxicology has expanded to the molecular level to investigate these effects. Much of the research has been funneled towards understanding the contribution and effects of PM chemical constituents on activating pro-inflammatory pathways in human bronchial epithelia. Given the complexity of PM and the abundance of highly variable and relative constituents, it is difficult to determine the mechanisms involved in activating inflammatory canonical pathways. Lonkar and Dedon (2011) hypothesized that PM_2.5_ constituents are predominantly related to molecular mechanisms orchestrating inflammatory processes associated with lung diseases [13].

Studies conducted by Gualtieri et al. (2010) and Perrone et al. (2013) report the presence of inorganic constituents (sulfates, nitrates, ammonium, chloride, and trace metals) contributing to inflammation via oxidative stress [14,15]. Nevertheless, the consensus indicates that air pollution-induced health effects involve an inflammation-related cascade occurring in various critical key tissues (lung, vascular, and heart) in association with oxidative stress [16,17,18,19]. These findings support the alternate hypothesis that PM constituents influence the initiation of an inflammatory mechanism via biochemical interactions between organic and inorganic constituents. We found that trace metals in airborne PM_2.5_ extracts greatly influence the cytotoxicity of some sites in Puerto Rico (Guayanilla). In contrast, the toxicity at other sites (Guayama and Ponce) greatly depends on their organic constituents [20]. Delfino et al. (2010) suggests that organic compounds, particularly polycyclic aromatic hydrocarbons (PAHs), majorly contribute to oxidative stress-inducing inflammatory pathways [21]. The orchestration of effects via biochemical interactions has yet to be determined. Efforts aiming at the chemical variability of PM, which may also vary with seasons, could explain or act as possible exogenous sources of oxidative stress [20,22,23].

Urban PM exposure elicits an ROS-mediated inflammation response in the human bronchial epithelium [24]. Various chemokines and cytokines play a significant role in initiating and controlling inflammation [25]. Among these, interleukin 1 (IL-1), interleukin 6 (IL-6), and tumor necrosis factor-alpha (TNF-α) are markers identified in lung tissue injury [25,26]. A substantial amount of evidence has been documented that PM constituents promote the secretion of pro-inflammatory mediators in non-antigen-presenting human bronchial epithelial cells [27,28,29,30,31,32,33]. This idea was initially thought to be characteristic of only antigen-presenting cells. There are several other signaling pathways suggested, including induction of the toll-like receptors, phosphatidylinositol 3-kinase, and nuclear erythroid 2-related factor 2 after PM exposure, which result in the enhancement of pro-inflammatory gene expression in airway epithelial cells through ROS-mediated activation of MAPK (ERK, JNK, p38 MAPK) and downstream NF-κB signaling pathways [24,25,26,27,28,29,30,31,32,33,34].

Many proposed mechanisms are extrapolated or expanded from classical molecular pathways from different experimental models. This work aims to better understand the toxicological effects of PM_2.5_ by using extracts (from Puerto Rico) throughout the years and exposing human bronchial epithelial cells (BEAS-2B). Here, we present evidence of several pro-inflammatory mediators being expressed after exposure to airborne PM_2.5_ in the absence of INFγ. The genetic expression profile and protein secretion of different key players in inflammation were studied. We hypothesize that exposure to PM extract allows the Class II trans-activator (CIITA) and the major histocompatibility complex class II (MHCII) expression in the absence of INFγ.

## 2. Materials and Methods

### 2.1. Particulate Matter Collection

PM_2.5_ samples were collected on Teflon PM_2.5_ filters using a Partisol-Plus Model 2025 Sequential Air Sampler (Rupprecht and Patashnick Co., Inc., Albany, NY, USA) by the Puerto Rico Environmental Quality Board (PR-EQB), which maintains a set of monitoring stations at various locations around the island as part of their monitoring program. Station EQB Bayamón (SEQB-B; +18.399820 (longitude), −66.171125 (latitude); AQS#72-021-0009) and Station EQB Humacao (SEQB-H; +18.153440, −65.828617; AQS #72-069-0001) samples for the same year (2013) were collected. SEQB-H represents a semi-rural environment, while the urban environment representation was referenced using SEQB-B. The same sampling apparatus and collection procedure were used for all stations. Filters from all stations were collected throughout the year and combined as a composite sample to obtain enough material for the experimental procedure.

### 2.2. Particulate Matter Extraction

All glassware was washed using a modified cleanup procedure [35]. Filters obtained from the PR-EQB were extracted in 50 mL of hexane/acetone 1:1 using a microwave extraction apparatus. We chose microwave-assisted extraction (MAE) due to the available literature regarding its use and the expanded optimization and validation of the methodology [36].

A closed microwave Teflon vessel extracted organic and inorganic compounds from composite filters. Several filters were introduced into a closed vessel containing enough solvent fluid (1:1 acetone: hexane) to submerge the filter. Then MAE was performed for 30 min, of which 5 min were employed on heating the closed vessel to 80 °C and afterward emission of microwaves at a power of 300 Ω. After extraction, the vessels cooled to room temperature before opening. Extracted filters were removed, and additional filters were submerged in the remaining extract solvent to accumulate filter extract from a composite sample. The resulting composite solvent was then filtered through a 0.22 µm glass filter, stored in a 50 mL flask, and evaporated in a chemical hood. Composite PM_2.5_ extracts were concentrated under a gentle nitrogen stream, transferred to a smaller pre-weighed amber vial, and evaporated until completely dry. Vials were weighed several times until a constant weight was obtained. All composite extracts were stored at −20 °C until further use. To have enough material to evaluate the toxicological responses of airborne PM_2.5_ extracts, combining extracts (1:1 combination of SEQB-B and SEQB-H) was necessary. We used 25 µg/mL of this PM_2.5_ composite to evaluate the various toxicological responses of cytokine secretion and gene expression assays.

### 2.3. Cell Culture and Exposure to PM_2.5_ Extract and Deferoxamine

Human bronchial epithelial cells (BEAS-2B) were obtained from the American Type Culture Collection (ATCC CRL-9609). Briefly, the cells were cultured according to the ATCC recommended protocol, maintained in bronchial epithelial basal medium (BEBM), and supplemented with epidermal growth factor, insulin, hydrocortisone, epinephrine, bovine pituitary extract, and transferrin (BEBM SingleQuot Kit, #CC-3170, Muenchensteinerstrasse 38, Basel, Switzerland). Cells were grown to 80% confluence in a T75 cm culture flask with nine passages. Cells were detached using a 0.25% trypsin-EDTA solution and centrifuged (cell passages of 7–9 were used). This allows for an approximate recovery of 1–3 × 10^6^ cells. Cells were seeded at a density of 1 × 10^4^ cells/well on a 96-well plate and incubated for 24 h to allow the attachment of the seeded cells. Cells were exposed to PM_2.5_ extracts at 25, 50, 75, and 100 μg/mL concentrations for 4 and 8 h. Each test was performed in duplicate for each extract concentration tested. These experiments were carried out using 0.01% DMSO as the carrier. Positive controls, such as LPS, and other controls, such as 0.01% DMSO, were simultaneously used with the experimental samples. After treatment, cell supernatants were decanted by inversion, and an MTT reagent was added to determine cell viability. The same protocol was used for similar experiments at the selected dose, and supernatants were collected for the remaining assays (gene expression and pro-inflammatory protein quantification). All recovered supernatants were stored at −80 °C until further analysis.

### 2.4. Cell viability (Cytotoxicity)

The MTT Cell Proliferation Assay (Abcam, Cambridge, UK) was employed to assess cell viability. The assay is based on converting a water-soluble MTT (3-(4,5- dimethylthiazol-2-yl)-2,5-diphenyltetrazolium bromide) compound to an insoluble formazan product. Treatment with PM_2.5_ extracts from SEQB-B, SEQB-H, and CuSO_4_ (treated at 250, 500, 750, and 1000 μM) were examined for cell viability using the MTT assay. Pure cell media and positive controls (Triton X-100, Sigma-Aldrich, Saint Louis, MI, USA) were concurrently assayed, with BEAS-2B cells incubated at different concentrations. After the 24 h exposure period, media containing the respective treatment were discarded, and 50 μL of serum-free media + 50 μL of MTT reagent was added to the wells. Cells were incubated at 37 °C for 3 h. Afterward, MTT solvent was added, and the plate was placed in a shaker for 15 min. Absorbance was measured at OD595 using an Ultramark microplate reader (Bio-Rad, Richmond, CA, USA). To determine the relative contribution of trace metals, two additional sets of cells (*n* = 3 dish) were also used to treat BEAS-2B with organic extracts and deferoxamine mesylate (DF, Sigma, cat # D9533), a metal chelator, at a final concentration of 50 μM Extracts with 50 μM DF were sonicated in a water bath for 30 min before cell exposure. DF (50 μM) was evaluated for cell viability and resulted in non-toxic to BEAS-2B. DMSO was used as a vehicle at a final concentration of 0.1%. Appropriate controls: medium, DMSO, and media exposed to DF were run simultaneously with each experiment. Blank filter extracts were also tested. A 24 h treatment with PM_2.5_ extracts from both stations, and CuSO_4_ (treated at 250, 500, 750, and 1000 μM) and cell viability were examined using the MTT assay [20]. Pure cell media and positive controls (Triton X-100) were concurrently assayed, with BEAS-2B cells incubated at different concentrations. After the exposure period, media containing the respective treatment were discarded, and 50 μL of serum-free media + 50 μL of MTT reagent was added to the wells. Cells were incubated at 37 °C for 3 h. Afterward, MTT solvent was added, and the plate was placed in a shaker for 15 min. Absorbance was measured at OD595 using an Ultramark microplate reader (Bio-Rad, Richmond, CA, USA).

### 2.5. Gene Expression

The expression of the following genes using their respective probes HLA-DRα Hs00219575_mL, CIITA Hs00931699_mL, and IRF-1 (Hs00971964_g1, all from Applied Biosystems, Waltham, MA, USA) were determined via quantitative RT-PCR. The method uses the Norgen-Biotek Total RNA Purification Kit (Norgen-Biotek^®^, Thorold, ON, Canada) to isolate and purify total RNA from cultured adherent BEAS-2B (P7-9). Purification was achieved by directly lysing the cells with 350 μL of Buffer RL in the culture plate. The purified RNA sample was stored at −70 °C for future use. Extracted RNA content was converted to cDNA using the iScript™ cDNA Synthesis Kit from Bio-Rad. After cDNA synthesis, the TaqMan Gene Expression Assay was used to measure the relative quantitation of the specific gene. This was conducted using a Step One Real-Time PCR System (Applied Biosystems, Waltham, MA, USA) and StepOne V2.3 Software. The relative change in gene expression was determined by using the ΔΔCT method. The GAPDH (Hs02786624_g1, Applied Biosystems, Waltham, MA, USA) housekeeping gene was used to normalize the target genes.

We analyzed the relative expression of HLA-DRα, CIITA (MHC Class II transcriptional regulator), and IRF-1 on BEAS-2B treated with PM_2.5_ extracts after 4 and 8 h of exposure. LPS (5 μg/mL) was used as a positive control, DMSO (0.01%) with the serum-free medium as a negative control, and for the samples (25 μg/mL of PM_2.5_ cocktail 1:1 combination of SEQB-B and SEQB-H).

### 2.6. Cytokine Assay

The levels of cytokines IL-6, IL-8, and INFγ were analyzed using a Human Cytokine A Premixed Magnetic Luminex Performance Assay (R&D Systems, Minneapolis, MN, USA) according to the manufacturer’s instructions. Cytokine concentrations were determined using the dual laser flow analyzer Luminex 100/200 (Luminex Corp., Austin, TX, USA). Standard curves for each cytokine were plotted using a 5-parameter logistic fit (5-PL). Each sample was run in duplicate. BEAS-2B was exposed at 25 μg/mL of PM cocktail (of SEQB-B and SEQB-H), and pro-inflammatory cytokines IL-6 and IL-8 were quantified, together with INFγ, to explore inflammation as a response to 4 and 8 h exposure with (0.01% DMSO in BEBM medium was used as controls).

### 2.7. STAT1 Phosphorylation

Invitrogen STAT1 (Phospho) [pY701] Human InstantOne ELISA kit was used at different time points (1, 2, and 4 h) to determine the activation of STAT1 via phosphorylation, depending on the treatment. The ELISA test consisted of 50 μL of cell lysate added to 50 μL of freshly prepared antibody cocktail after treatment in each experimental well. Plates were incubated for 1 hr. at room temperature and shaken at 300 rpm. Plates were rinsed three consecutive times with 200 μL/well wash solution followed by 100 μL/well of detection reagent and incubated for 10–30 min while shaken at 300 rpm. A 100 μL stop solution was added, and absorbance was immediately read at 450 nm. The positive control included cell lysate stimulated with EGF (1 µg/mL for 10 min) included with the kit. The negative control consisted of untreated cells, and p701-STAT1 phosphorylation was measured at 1–4 h. The sample size corresponds to three culture replicates.

To investigate the effect of trace metals in PM_2.5_ extract on the activation of STAT1 via phosphorylation, we added the chelating agent deferoxamine mesylate (50 μM) to the extract before treatment. Data were normalized using the average absorbance of the controls. Y701-STAT1 phosphorylation was measured at 1–4 h. Treatment consisted of 25 μg/mL of urban airborne PM_2.5_ composite extract from SEQB-B and SEQB-H mixed with deferoxamine mesylate (50 μM).

### 2.8. Statistical Analysis

Results are shown as mean values and the corresponding standard deviations. Statistical analysis was performed using Sidak’s multiple comparison test, two-way ANOVA, and Tukey’s multiple comparison tests as deemed appropriate, using GraphPad Prism 8 (V 8.0.2; GraphPad Software Inc., San Diego, CA, USA). Significant differences were reported considering *p*-values of <0.05.

## 3. Results

### 3.1. Evaluating CuSO_4_ and PM_2.5_ Composite Extract on Human Bronchial Epithelial Cells

**Figure 1:**
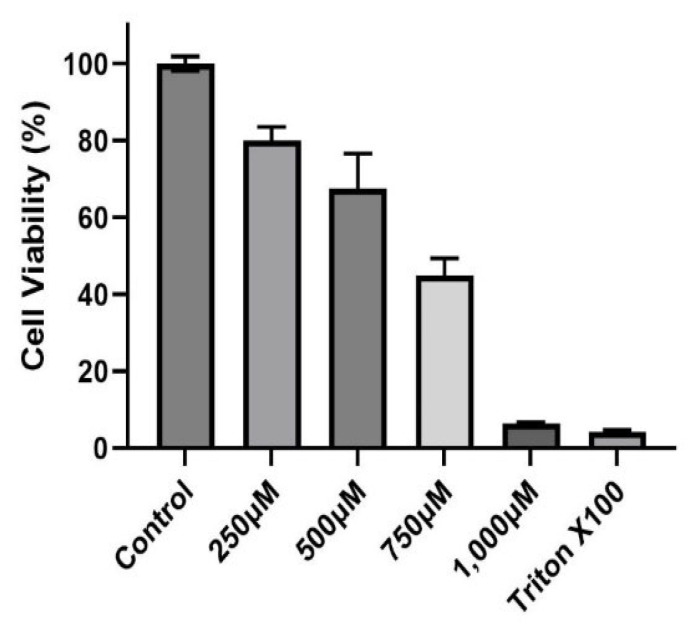
*Cytotoxicity of Copper Sulfate (CuSO_4_) on BEAS-2B cells.* Cells were exposed for 24 h at different concentrations (250, 500, 750, and 1000 μM) of CuSO_4_. Cell viability was determined using the MTT assay. Error bars are the standard error of the mean. LC_50_ was estimated at 755.73 μM using the AAT Bioquest Calculator. (*n* = 3).

**Figure 2:**
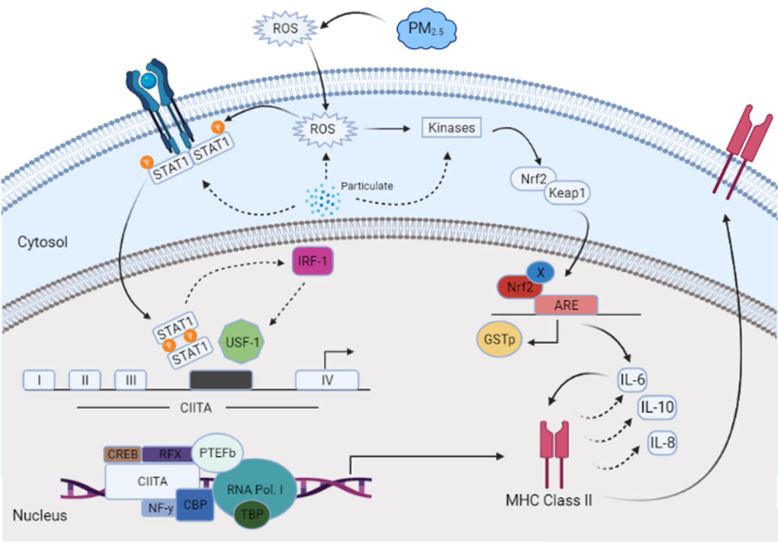
A conceptual model with the proposed downstream effectors involved the induction of CIITA and MHCII genes affected by airborne PM_2.5_ constituents in human lung cells (BEAS-2B).

**Figure 3:**
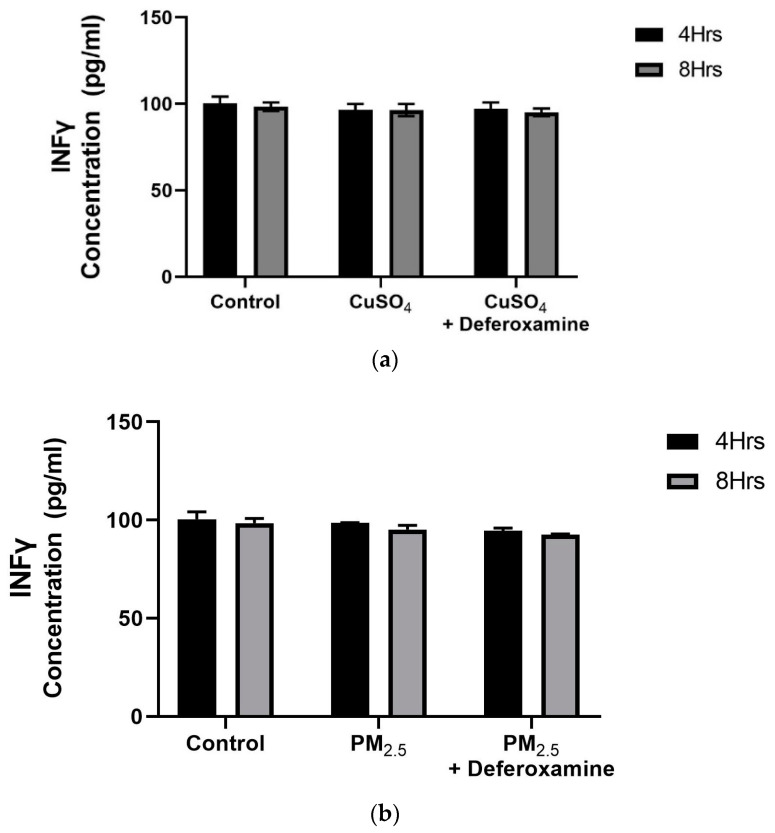
*Effects of CuSO_4_ (**a**) and PM_2.5_ (**b**) exposure on IFNγ secretion by BEAS-2B epithelial cells.* (**a**) Cells were exposed for 4 and 8 h at 250 μM CuSO_4,_ and (**b**) 25 μg /mL of PM_2.5_ (SEQB-B and SEQB-H composite extract), cell supernatants were evaluated for IFNγ secretions at these time points. (**a**) The control consisted of cell supernatants supplemented with BEBM and (**b**) cell supernatants from media supplemented with 0.01% DMSO. In addition, a deferoxamine chelation treatment was performed at 4 and 8 h to evaluate if copper or heavy metals affected IFNγ secretion. IFNγ levels were analyzed using a Human Cytokine A Premixed Magnetic Luminex Performance Assay (Luminex 200). Assay Supernatant samples were analyzed in duplicate (*n* = 2). Error bars illustrate the standard error of the mean.

A dose–response experiment was conducted to select the proper dose for our (CuSO_4_) experimental design. At a concentration of 250 µM (CuSO_4_), more than 80% cell viability remained (Figure 1), therefore, this concentration was used in further experiments. The mechanistic pathway involved with MHCII gene expression has been elucidated and known to be mediated through the INFγ receptor, which initiates the induction of the trans-activator CIITA [37,38,39,40] and master regulator of MHCII genes. We have constructed a simplified diagram illustrating the major effector links downstream of this pathway (Figure 2). We found that IFNγ levels remain stable during CuSO_4_ and PM_2.5_ regardless of the exposure time evaluated (0–8 h). No significant change was observed in IFNγ levels after treatment with the metal chelator deferoxamine at the time points studied (Figure 3a,b). Therefore, we conclude that neither PM_2.5_ nor CuSO_4_ at the dose tested influence INFγ secretion. These results indicate that CuSO_4_ exposure and PM_2.5_ at the concentrations tested do not cause an increase or reduction in IFNγ secretion at these time points. Therefore, neither CuSO_4_ or PM_2.5_ exposure are significant key players in the early stages by activating or inducing IFNγ secretion and influencing CIITA or MHCII gene responses through its IFNγ receptor pathway. Since IFNγ levels are important for the immune response of the CIITA and MHCII genes, we also evaluate cytokine expression at 250 µM (CuSO_4_).

### 3.2. Effect of CuSO_4_ Exposure on Immune Function

#### 3.2.1. CIITA and HLA-DRα Immune Responses

**Figure 4:**
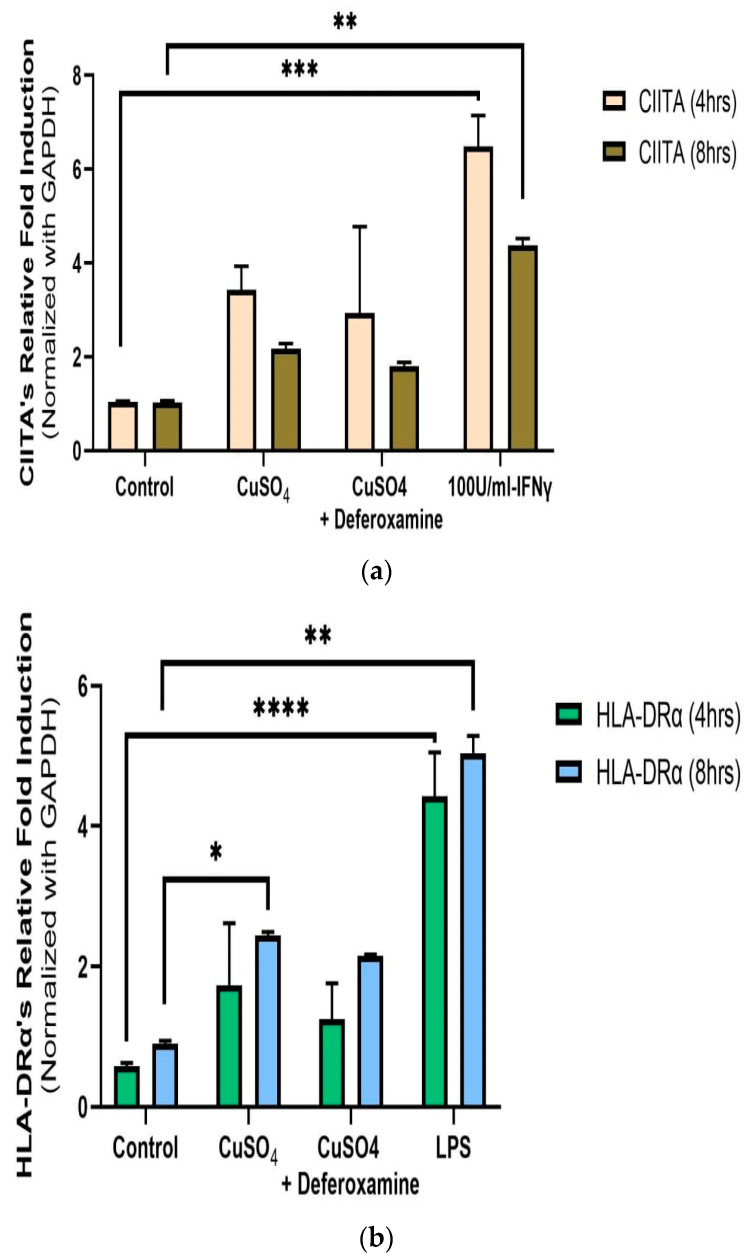
*Effects of CuSO_4_ exposure on CIITA (**a**) and HLA-DRα (**b**) gene expression (average mRNA levels at 4–8 h) in BEAS-2B.* Cells were exposed for 4–8 h to 250 μM CuSO_4,_ and mRNA levels were determined. The control consisted of cells cultured in media supplemented with BEBM, (**a**) IFNγ and (**b**) LPS at 5 μg/mL as a positive control. Chelation (deferoxamine) treatment was performed at 4 and 8 h. Samples were assayed in duplicate. Error bars illustrate the standard error of the mean. Dunnett’s multiple comparison tests were performed to assess statistical differences at a 0.05 significance level. (**a**) ** *p*-value < 0.0096, *** *p* = 0.0004; (**b**) * *p*-value = 0.0301; ** *p*-value = 0.0015; **** *p*-value < 0.0001.

Relative measurements of mRNA expression of the related immune genes CIITA and HLA-DRα at 4 and 8 h after 250 μM CuSO_4_ treatment were performed in human lung cells (BEAS-2B). No significant differences in average mRNA levels of CIITA were observed after 4 or 8 h (Figure 4a). Although no significant difference at the 95% interval was observed, the levels of CIITA mRNA were slightly higher at both time points. The mRNA levels for MHCII expression were significantly higher at 8 h (Figure 4b). At 250 μM of CuSO_4,_ no induction of either IFNγ, CIITA, or MHCII at 4 h occurs.

#### 3.2.2. STAT1 p701 Phosphorylation, and IRF-1 mRNA Expression in BEAS-2B Cells

**Figure 5:**
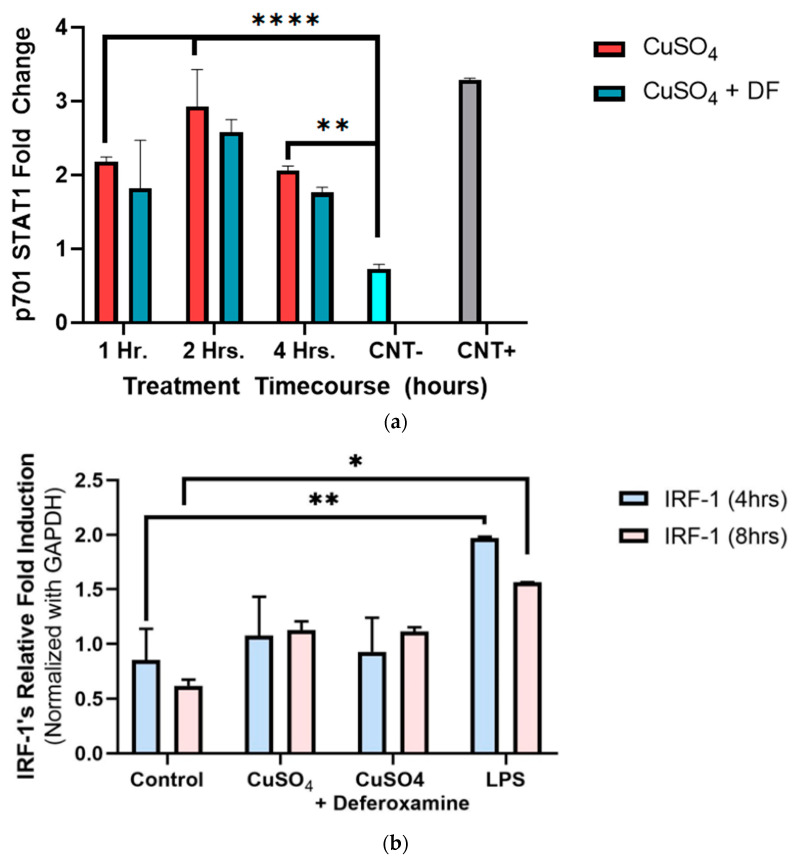
*Effects of CuSO_4_ and chelating agent (deferoxamine) on STAT1 p701 and IRF-1 mRNA levels of treated BEAS-2B cells.* BEAS-2B cells were exposed for (**a**) 1 h, 2 h, and 4 h and (**b**) 4–8 h to 250 μM of CuSO_4_ pentahydrate and 250 μM of CuSO_4_ deferoxamine. The negative control -CNT consisted of cell lysis buffer 1×, and the positive control (CNT+) of A545 treated with 10 μg/mL of EGFR. (**b**) Control consisted of cells cultured in media supplemented with BEBM and LPS (5 μg/mL) was used as a positive control. Samples were performed in triplicate. Error bars illustrate the standard error of the mean. Colorimeter reads were repeated five times and averaged. For (**a**) ** *p*-value < 0.002, **** *p*-value < 0.0001, (*n* = 2). (**b**) Samples were performed in duplicate (*n* = 2). Dunnett’s multiple comparison tests assessed statistical differences at a 95% confidence interval. * *p* = 0.0063, ** *p* = 0.0022.

To better understand and define our conceptual model, Y701-STAT1 phosphorylation was measured at 1–4 h of CuSO_4_ exposure in BEAS-2B lung cells_._ There was a two-fold increase in Y701 phosphorylation of STAT1 as early as 1 h when compared to the negative control, reaching a maximum induction peak at 2 h and returning to the induced 1 h levels after 4 h of Cu exposure (Figure 5a). The metal chelator of deferoxamine mesylate did not significantly alter the phosphorylated tyrosine levels obtained at treatment time points (1–4 h). IRF-1 exposure to 250 μM CuSO_4_ at 4 h showed no significant expression (Figure 5b). However, a two-fold increase in IRF-1 mRNA expression after 8 h was evident. The addition of deferoxamine mesylate did not alter IRF-1 mRNA levels.

#### 3.2.3. Quantification of Pro-Inflammatory Cytokines after CuSO_4_ Treatment in BEAS-2B

**Figure 6:**
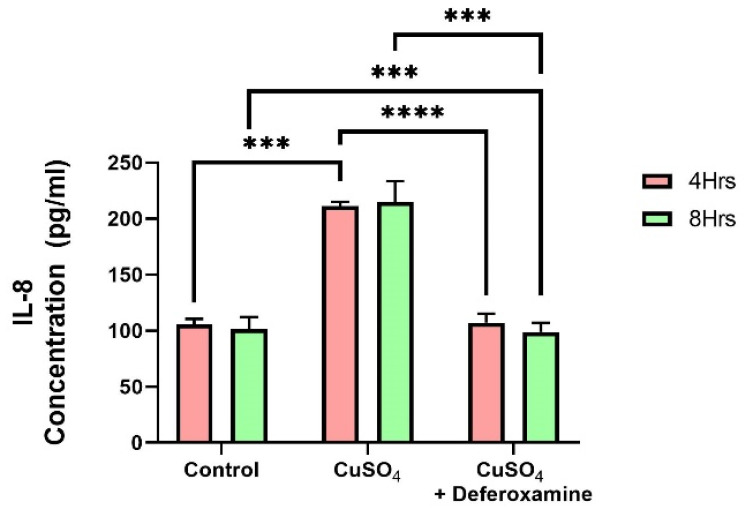
*Effect of CuSO_4_ exposure on IL-8 secretion by BEAS-2B.* Cells were exposed for 4 and 8 h to 250 μM CuSO_4_. The control consisted of supernatant from cells grown in BEBM. A deferoxamine chelation treatment was performed at 4 and 8 h to evaluate the effect of copper on IL-8 secretion. Samples were performed in duplicate. Error bars illustrate the standard error of the mean. A two-way ANOVA test assessed statistical differences at a 0.05 significance level. *** *p*-value = 0.0001; **** *p*-value < 0.0001.

A vast amount of research has been performed on trace element exposure and the development of inflammatory responses. A recent review reveals epidemiological evidence on the effect of heavy metal exposure on childhood immune function from multiple perspectives [41]. Cu can also catalyze the generation of reactive species/radicals, potentially damaging macromolecules: Proteins, lipids, and nucleic acids [42]. In excess, it can replace many divalent elements (zinc, iron, magnesium, and cobalt) in various metalloproteins [43]. It is known that bronchial epithelial cells can secrete pro-inflammatory cytokines such as IL-6 and IL-8, CIITA, and MHCII [30,31,32,44]. Therefore, we studied the effect of CuSO_4_ on IL-6 and IL-8 secretion on BEAS-2B. These two cytokines (IL-6, IL-8) were examined at 4 and 8 h after CuSO_4_ exposure at (250 μM). No significant changes were observed in IL-6 secretions after 4 or 8 h of exposure to 250 μM CuSO_4_ or after deferoxamine treatment. However, IL-8 secretions increased as much as two-fold after 4 h of CuSO_4_ treatment (Figure 6). Deferoxamine treatment did not significantly decrease the levels of IL-8 either at 4 or 8 h. Thus, CuSO_4_ treatment selectively induces cytokine secretion, probably related to the transcription factors that regulate their expression.

### 3.3. Effect of PM_2.5_ Exposure on Immune Function

#### 3.3.1. CIITA and HLA-DRα Immune Responses

**Figure 7:**
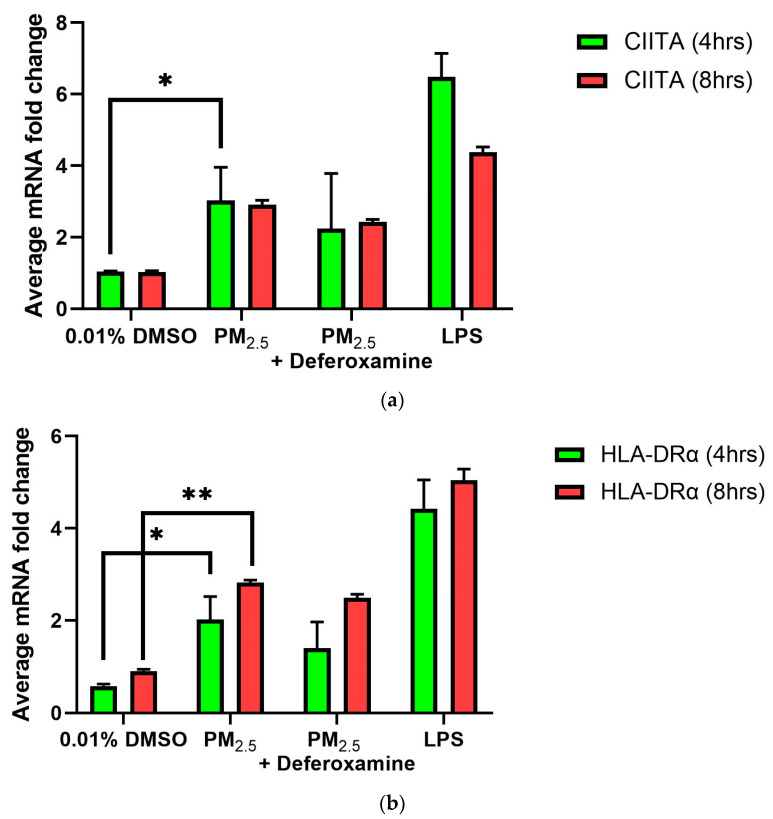
*Effect of PM_2.5_ exposure on CIITA (**a**) and HLA-DRα* (***b***) *gene expression (average mRNA) levels in BEAS-2B cells*. Cells were exposed for 4 and 8 h at 25 μg/mL of PM_2.5_ extracts, and mRNA levels were determined. PM_2.5_ samples comprised both SEQB-B and SEQB-H (1:1). Control consisted of cells cultured in media supplemented with 0.01% DMSO. Deferoxamine chelation treatments were performed at 4 and 8 h to evaluate the effect of PM_2.5_ extract trace metal constituents on CIITA and HLA-DRA gene expression. Samples were performed in duplicate. Error bars illustrate the standard error of the mean. Dunnett’s multiple comparison test was performed to assess statistical differences at a 0.05 significance level. (**a**) * *p*-value = 0.0460; (**b**) * *p*-value = 0.0103, ** *p*-value = 0.0060.

As previously presented, exposure to *PM_2.5_* extract or *CuSO_4_* did not alter the immune response of IFNγ secretion by human lung cells (Figure 3a,b). Therefore, we evaluate the immune functions of interest, such as *CIITA* and *HLA-DRα*. The averaged mRNA of CIITA after *PM_2.5_* (SEQB-B and SEQB-H composite extract) exposure to BEAS-2B resulted in a two-fold increase after 4 h Yet, there is no significant difference using deferoxamine (Figure 7a). Treatment after 8 h remained like that at 4 h (no significant difference between these two points), with statistical significance compared to the baseline levels (*p* < 0.001). A two-fold increase in the average mRNA levels of HLA-DRα after PM exposure at 4 h was noticeable (Figure 7b).

Adding deferoxamine mesylate to the PM cocktail before treatment resulted in a 50% reduction in HLA-DRα mRNA levels at 4 h At 8 h, there was a slightly higher increase in mRNA HLA-DRα levels (2.5-fold increase), but no statistically significant differences were determined compared to the levels obtained at 4 h or after deferoxamine treatment. *CIITA and HLA-DRα immune responses* were very similar, as expected since CIITA is the master regulator of HLA-DR*α* [45].

#### 3.3.2. Effects on pY701, STAT1, and Deferoxamine Mesylate by Urban SEQB-B and Rural SEQB-H Airborne PM_2.5_ Extract

**Figure 8:**
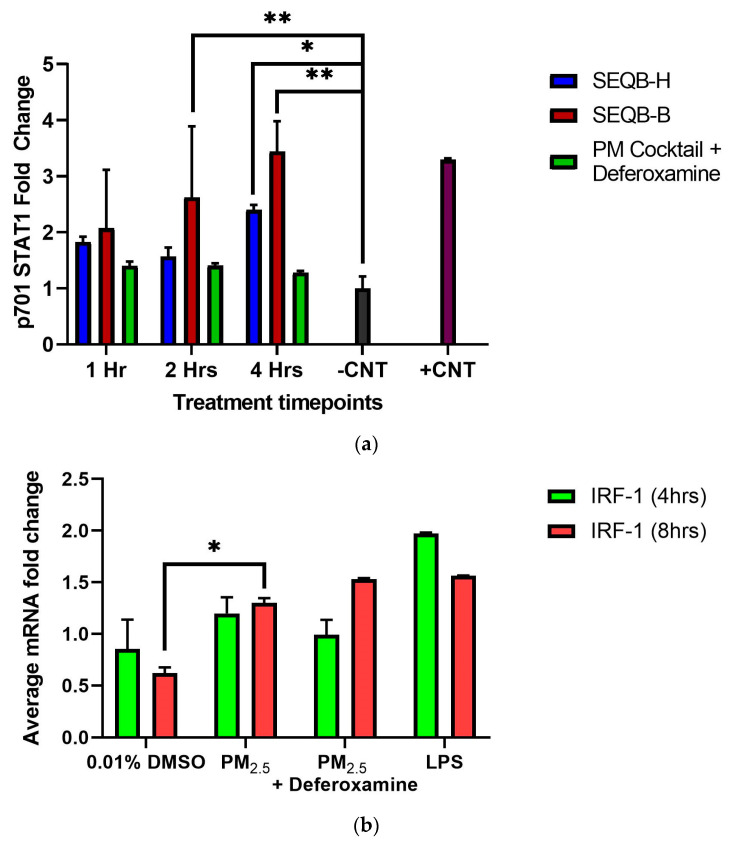
*Effect of PM_2.5_ extract exposure on STAT1 p701 (**a**) and IRF-1 (**b**) (average mRNA) levels in BEAS-2B cells.* Cells were exposed for (**a**) 1, 2, and 4 h and (**b**) for 4 and 8 h at 25 μg/mL of PM_2.5_ extracts. The control consisted of cells without PM_2.5_ extract and positive control of A545 cell lysate treated with 10 μg/mL of epidermal growth factor receptor. A deferoxamine chelation treatment was performed at each time interval to evaluate the effect of trace metals on STAT1 phosphorylation and IRF-1 gene expression by PM_2.5_ extract constituents. Samples were performed in duplicate. Error bars illustrate the standard error of the mean. Dunnett’s multiple comparison test was performed to assess statistical differences at a 0.05 significance level (**a**) ** *p*-value < 0.0100, * *p*-value = 0.0271 and (**b**) * *p*-value = 0.0018.

It is known that INFγ affects the phosphorylation of STAT1, which influences the expression of other genes, such as CIITA and, subsequently, the MHCII genes [46,47]. Since we showed that INFγ secretions at 4 or 8 h were not altered by PM_2.5_ treatment, yet CIITA and HLA-DRα expression were increased, the state of STAT1 of tyrosine 701 phosphorylation was examined in epithelial cells exposed to 25 µg/mL of. STAT1 tyrosine 701 phosphorylation was induced at 2 and 4 h (Figure 8a). This is true for both PM_2.5_ extracts.

A clear trend shows a direct effect on STAT1 (1 < 2 < 4 h)-phosphorylated Y701 tyrosine by PM_2.5_ exposure compared to its respective control. A similar effect with SEQB-H PM_2.5_ was also observed (yet not as strong as with the urban dust), with the greatest difference at 4 h (Figure 8a).

At 2 h after exposure, an approximate 20% increase in STAT1 phosphorylation was observed by PM_2.5_ (SEQB-B) extract. Compared to the control, phosphorylated tyrosine residues of STAT1 show a three-fold difference at 4 h. This change also significantly differs from 1 h (*p* = 0.003; *p* = 0.002, respectively). SEQB-H extract induced only 1-fold p701 phosphorylation of STAT1 after 1 h, with a minor change at 2 h. At 4 h, however, there was a 1.3-fold change, which was statistically significant (*p* = 0.003). Deferoxamine mesylate did not show any significant effect on phosphorylated tyrosine at either 1 or 2 h (although there seems to be a difference in SEQB-B at 2 h; however, there is much variability). Nevertheless, a marked reduction of approximately 50% in the p701 phosphorylation for SEQB-B at 4 h was observed due to chelation (Figure 8a). PM_2.5_ from urban areas has the greatest effect on p701 phosphorylation and increases with time. Heavy metals are associated with this increase. However, rural PM_2.5_ does not show this direct increase with time. A significant increase in IRF-1 mRNA levels was observed after PM_2.5_ exposure at 4 or 8 h, yet deferoxamine mesylate treatment did not affect the expression of the gene (Figure 8b).

#### 3.3.3. Pro-Inflammatory Cytokines IL-6 and IL-8 after PM_2.5_ Exposure and Deferoxamine Mesylate Treatment

**Figure 9:**
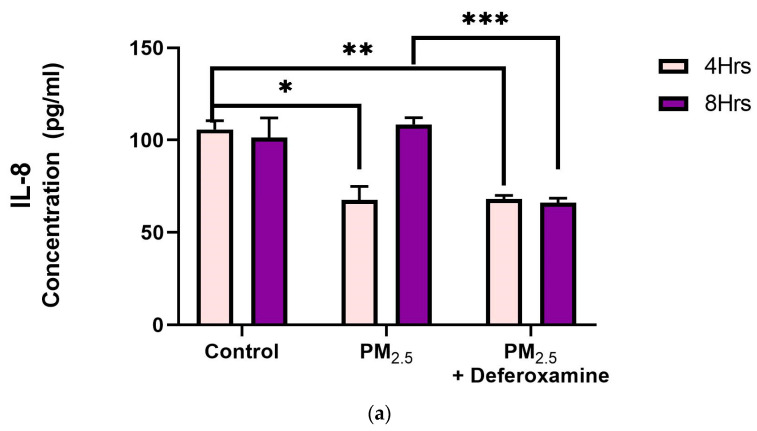

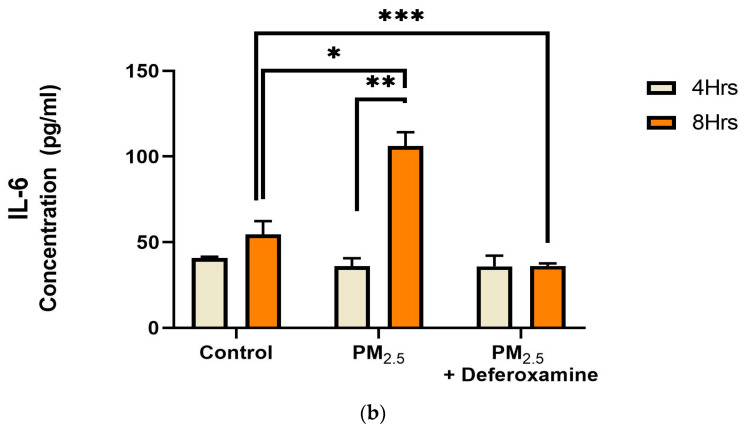
*Effect of PM_2.5_ exposure on IL-8 (**a**) and IL-6 (**b**) secretion by BEAS-2B.* Cells were exposed for 4 and 8 h at 25 μg/mL PM_2.5_ extract = SEQB-B and SEQB-H 1:1 composite sample. The control consisted of a media supernatant from cells grown in 0.01% DMSO. A deferoxamine chelation treatment was performed at 4 and 8 h to evaluate the effect of PM_2.5_ trace metal constituents on IL-8 and IL-6 secretion. Samples were performed in duplicate. Error bars illustrate the standard error of the mean. (**a**) Tukey’s multiple comparison tests assessed statistical differences at a 0.05 significance level for IL-8. From left to right: * *p*-value = 0.0014, ** *p*-value = 0.0026, and *** *p*-value = 0.0003. (**b**) Sidak’s multiple comparison tests assessed statistical differences at the 95% confident interval level. * *p*-value = 0.0416, ** *p*-value = 0.0002, *** *p*-value < 0.0001 for IL-6.

IL-8 levels were significantly reduced after 4 h of PM_2.5_ treatment, yet deferoxamine treatment did not alter the response at this time point (Figure 9a). However, after 8 h, IL-8 levels were not altered as they were at 4 h compared to the control, suggesting that IL-8 levels returned to their normal level. Deferoxamine *mesylate* treatment at 8 h significantly reduced the IL-8 response (Figure 9a). Secreted levels of IL-6 did not show any significant response after 4 h of PM_2.5_ treatment (Figure 9b). Yet, a two-fold increase was observed after 8 h, suggesting a delayed response for IL-6 and susceptibility to deferoxamine treatment. This response is like that observed with IL-8, where deferoxamine reduces both cytokine levels (IL-6 and IL-8) after 8 h.

## 4. Discussion

Longitudinal studies on air quality in Puerto Rico have shown that there has been a gradual improvement in air quality, with particulate matter decreasing over time [31,35]. Nonetheless, the fraction composition of *PM_2.5_* varies with the season, climate changes, and location, with natural and anthropogenic chemical constituents contributing to ambient air quality. Toxicologists have thoroughly studied the latter due to its importance and significance in enriching *PM_2.5_* with inorganic and organic constituents. Exposure to PM_2.5_ constituents from Puerto Rico, such as organics and toxic metals, has been shown to induce pro-inflammatory responses in human bronchial epithelial cells [28,34]. BEAS-2B cells respond to dose-scaling and time-dependent processes differently, varying with both the cell type and the cell culture conditions, thus being efficient using in vitro cell models to help elucidate toxicological mechanisms [48,49].

It has been demonstrated that *PM_2.5_* constituents influence the initiation of inflammatory processes via biochemical interactions between organic and inorganic constituents and cell components. It is also known that reactive oxygen species (ROS) are generated as by-products of cellular metabolism through the electron transport chain (ETC) in mitochondria and via the cytochrome P450 metabolism. Metals associated with *PM_2.5_* can generate oxidative damage in lung tissue by inducing the formation of free radicals and ROS [50]. ROS can serve as both intra- and inter-cellular messengers and participate in the activation of cell signaling cascades and gene expression [51,52]. We have previously reported that airborne *PM_2.5_* extract from Puerto Rico stimulates oxidative stress and induces inflammatory responses in human lung cells through Nrf2 [32]. We have also reported that copper (Cu) is a significant component of *PM_2.5_* from various locations in Puerto Rico [53,54,55,56]. Since Cu is found in *PM_2.5_* from Puerto Rico and *PM_2.5_* induces ROS, which in turn is associated with the secretion of pro-inflammatory mediators, we, therefore, performed a series of experiments to test the effects of copper sulfate (CuSO_4_) and *PM_2.5_* on immune response and present the results of these experiments separately.

A paramount in vitro study has been published in respiratory toxicology, cardiovascular disease, and immunotoxicology, focusing on the mechanisms behind PM_2.5_ exposure related to adverse health effects. However, the complexity behind PM_2.5_ constituents has made it difficult to unveil the specific compounds or combinations responsible for orchestrating these adverse health effects. Therefore, elucidating the mechanisms leading to the exacerbation of inflammatory responses and how these relate to and interconnect with PM_2.5_ exposure and metabolic pathways remains a fertile path that challenges and contributes to critical toxicological knowledge. It has been postulated for decades that the anti-glucocorticoid pregnenolone-16-alpha-carbonitrile (PCN) is a probe for hepatic defenses activated under environmental “stress” [57].

The content of cytochrome P-450 was found to double in cellular subfractions treated with PCN [58]. PCN, an anti-glucocorticoid, and glucocorticoids like dexamethasone (Dex) stimulate hepatic metabolism and the elimination of xenobiotics by binding to the nuclear Pregnane X Receptor (PXR), which then interacts with a distinct DNA response element associated with the induction of CYP3A genes [59,60,61,62,63]. It has also been reported that PCN and Dex elicit MHCII gene expression in rat livers [64]. Knowing that MHCII genes are expressed as a possible defense mechanism after exposure to xenobiotics (since PCN induces it), it was discovered that human lung epithelial cells (EC) were capable of inducing MHCII (HLA-DRα) and down-regulating CYP3A after 24 h exposure to PM_2.5_ extracts [28]. ECs may have primary immune functions that affect the balance between tolerance and inflammation, as evidenced by the expression of MHC class II on the EC surface, an area reported several decades ago [65,66]. The mechanistic pathway involved with MHCII gene induction has been known for many years to respond to the presence of INFγ, which is mediated by the transactivator CIITA [37,38,39,40].

Constitutive levels of MHCII both in the bronchial and alveolar epithelium, specifically in type II pneumocytes and ciliated ECs, have been previously reported [67,68,69]. Therefore, this finding and the fact that bronchial epithelial cells induce an MHCII response by PM_2.5_ extract prompted further studies to elucidate the molecular pathways involved in particle pollution. Our initial approach evaluated the secretion of INFγ in lung epithelial cells exposed to PM_2.5_ extracts and CuSO_4_ and found hardly any change (Figure 3a,b) in this cytokine with time [28].

To better understand inflammatory responses to PM_2.5_ exposure, we continued to evaluate MHCII induction in basal levels of INFγ and, hence, its association with its canonical inflammatory pathway using human bronchial epithelial cells. The effects on these mechanisms are thought to begin early and are initiated by oxidative stress events [14,70,71]. It is also known that toxic metals are associated with increased oxidative species (e.g., ROS and RNS), and previous work using PM_2.5_ extracts demonstrated the induction of pro-inflammatory responses in human lung cells [32]. *Here we show that PM_2.5_ initial effect on IL8 reduction and IL6 is due to organic constituents while subsequent induction effect at 8 h is due to trace elements (Figure 9a,b).*

The effects of PM_2.5_ are orchestrated by a myriad of chemical combinations consisting of inorganic and organic mixtures. Communities living in high PM_2.5_ areas with high nickel content, vanadium, and elemental carbon were found to have a higher risk of hospitalizations associated with cardiovascular disease [72]. Another epidemiological study provides evidence that the organic-related compounds from traffic emission sources, particularly PAHs, are associated with increased systemic inflammation [21].

Above, we discuss and convey that PM_2.5_ extracts induce MHCII in lung cells, and since CIITA initiates transcription of MHCII genes, most likely via effectors of the INFγ activated transcription pathway, we hypothesize an alternate regulatory pathway. We demonstrate that INFγ remains close to baseline levels, irrespective of the exposure time for either PM_2.5_ extract or *CuSO_4_*. Nevertheless, phosphorylation of the Y701 tyrosine of STAT1 (activation of STAT1; see diagram in Figure 2) occurs in the range of 1 to 4 h of cell exposure (two- to three-fold increase, respectively). This indicates that PM_2.5_ extract and *CuSO_4_* treatment significantly alter STAT1 phosphorylation without inducing significant changes in INFγ levels. This is shown to be possible by various routes, one of which was demonstrated by Ruvolo et al. (2003) through the induction of a posttranscriptional activator nuclear protein (SM) without directly stimulating the secretion of interferon-alpha/beta, an intermediate step in the regulatory pathway [73]. Likewise, IFN-independent activation of IFN-dependent signaling pathways such as Jak/Stat has also been documented, as seen during infection of monocytes via rerouting of IL-6 signaling [74]. A possible route by which this may happen is via the expression of IL-6, which activates JAK in the absence of STAT3 and can phosphorylate STAT1 [75]. Therefore, IL-6 could activate STAT1 without interferon [76,77,78,79]. The effect on the phosphorylation of STAT1 may be due to the organic content of PM_2.5_ since adding deferoxamine mesylate to the extract did not significantly reduce its level until 4 h after exposure. Perhaps no significance was found due to the large variability in phosphorylation at 1 h. Nevertheless, metals, such as Cr (IV), may induce STAT1 [80]. Heavy metals in PM_2.5_ extracts increased ROS after 1 h in BEAS-2B [32]. Therefore, we hypothesize that increased STAT1 induction could be due to increased ROS formation. Inorganic components can catalyze different oxidation reactions through their redox potential, whereas organic pollutants can produce reactive oxygen species (ROS) mainly in a stoichiometric process [81,82].

Since STAT1 phosphorylation is associated with regulating CIITA (the MHCII master regulator), we examined the mRNA levels of CIITA and found a three-fold increase compared to 4–8 h—control (Figure 7a). The MHCII gene’s mRNA (HLA-DRα) also increases after 4 h (two-fold expression), followed by a further increase at 8 h. Fuentes-Mattei et al. 2010 reported that MHCII remained relatively low at 12 h after PM_2.5_ exposure and increased four-fold at 24 h. We demonstrate that after PM_2.5_ exposure, CIITA mRNA levels increased at 4 h and remained elevated at 8 h; as did MHCII (*HLA-DRα*) levels. This agrees with previous findings where the bimodal expression of MHCII is reported in epithelial ovarian cancer cells [83]. Therefore, the increase in CIITA levels correlates with the increase in the MHCII gene as expected, however, this increase does not seem to be entirely dependent on the metal load since DF treatment did not reduce the expression of these genes (Figure 7). It is expected that low INFγ levels correlate with low IRF-1 expression. Normally, IRF-1 will respond to the presence of INFγ and recruit USF-1, which, together with STAT1, promotes transcription (see diagram, Figure 2) of CIITA [80]. Measurements of IRF-1 expression after PM_2.5_ extract treatment at the time points studied show an increase at 8 h (Figure 8b). The data indicate that the expression of CIITA and MHCII genes at these early time points is loosely related to inorganic constituents found in the PM_2.5_ extract, supporting *the hypothesis that organic constituents play a stronger role in the expression of these genes in lung EC*.

Fuentes-Mattei et al. (2010) had previously shown that PM_2.5_ polar organic extracts induced the secretion of IL-6 while significantly inhibiting IL-8 secretion, followed by up-regulation of MHCII expression after 24 h Other research showed IL-6 induction by PM_2.5_ extract at 8 h (Rodriguez-Cotto et al., 2015) [28,32]. We found IL-6 induction at 8 h and IL-8 inhibition at 4 h by PM_2.5_ extracts (Figure 9a,b) which is in full agreement with what was previously reported. However, this induction of IL-6 at 8 h and recovery in IL-8 at 8 h were found to be strongly dependent on heavy metals.

IL-6 could influence the signaling induction of STAT1 via an INFγ-like response, as previously discussed, and could also explain the bimodality observed with CIITA and MHCII responses, as presented here. The addition of deferoxamine mesylate did not cause any changes in IL-6 expression at 4 h but brought it below control levels after 8 h of exposure. Previous work demonstrates that PM_2.5_ induces ROS and, subsequently, nuclear erythrocyte factor 2 (Nrf2), which induces IL-6 [32]. Our data suggest bronchial epithelia can respond quickly to mitigate potential long-term cell damage. As presented here, bronchial epithelia activate a pro-inflammatory mechanism via an INFγ-independent activation of STAT1. Nonetheless, more specific work must be performed to mechanistically characterize a source-oriented starting point for this induction, as particulate matter composition is too variable.

## Data Availability

The data supporting this study’s findings are available on reasonable request from the corresponding author (B.Jiménez). The data are not publicly available due to content or information that could compromise research participant privacy or anonymity.
